# Analysis of a hit‐and‐run tumor model by HPV in oropharyngeal cancers

**DOI:** 10.1002/jmv.28260

**Published:** 2022-11-04

**Authors:** Danyelle Assis Ferreira, Adi Idris, Nigel A. J. McMillan

**Affiliations:** ^1^ Griffith Centre for Cell and Gene Medicine, Menzies Health Institute Queensland, School of Pharmacy and Medical Science Griffith University Southport Queensland Australia

**Keywords:** E7, human papilloma virus, oropharyngeal cancer

## Abstract

Several viruses are known to be associated with the development of certain cancers, including human papilloma virus (HPV), an established causative agent for a range of anogenital and head and neck cancers. However, the causality has been based on the presence of the virus, or its genetic material, in the sampled tumors. We have long wondered if viruses cause cancer via a “hit and run” mechanism such that they are no longer present in the resulting tumors. Here, we hypothesize that the absence of viral genes from the tumor does not necessarily exclude the viral aetiology. To test this, we used an HPV‐driven oropharyngeal cancer (OPC) tumor model and CRISPR to delete the viral oncogene, E7. Indeed, the genetic removal of HPV E7 oncogene eliminates tumors in vivo. Remarkably, E7 deleted tumors recurred over time and develop new mutations not previously seen in HPV^**+**^ OPC tumors. Importantly, a number of these new mutations are found to be already present in HPV^−^ OPC tumors.

## 1 INTRODUCTION

Around one‐third of cancers are caused by infectious organisms^1^ but this number may be much larger if viruses are able to initiate cancers but not be necessary for their ongoing growth. Such a mechanism has been speculated to occur via a “hit‐and‐run” phenomenon, a long‐theorized mechanism through which a viral infection promotes carcinogenesis (see review by Ferreira et al.)^2^ but is not required long‐term due to further oncogenic mutations that maintain the cancer in the absence of the original virus infection. Fifty years ago, this theory was first used to suggest the carcinogenesis of herpesviruses, particularly herpes simplex virus 2.^3^ Further speculation of the involvement of the hit‐and‐run mechanism has been reported for adenovirus,^4^ polyomavirus^5^ and gamma herpesviruses.^6^ However, conclusive biological evidence of hit‐and‐run as a carcinogenic mechanism has remained elusive.

**Figure 1:**
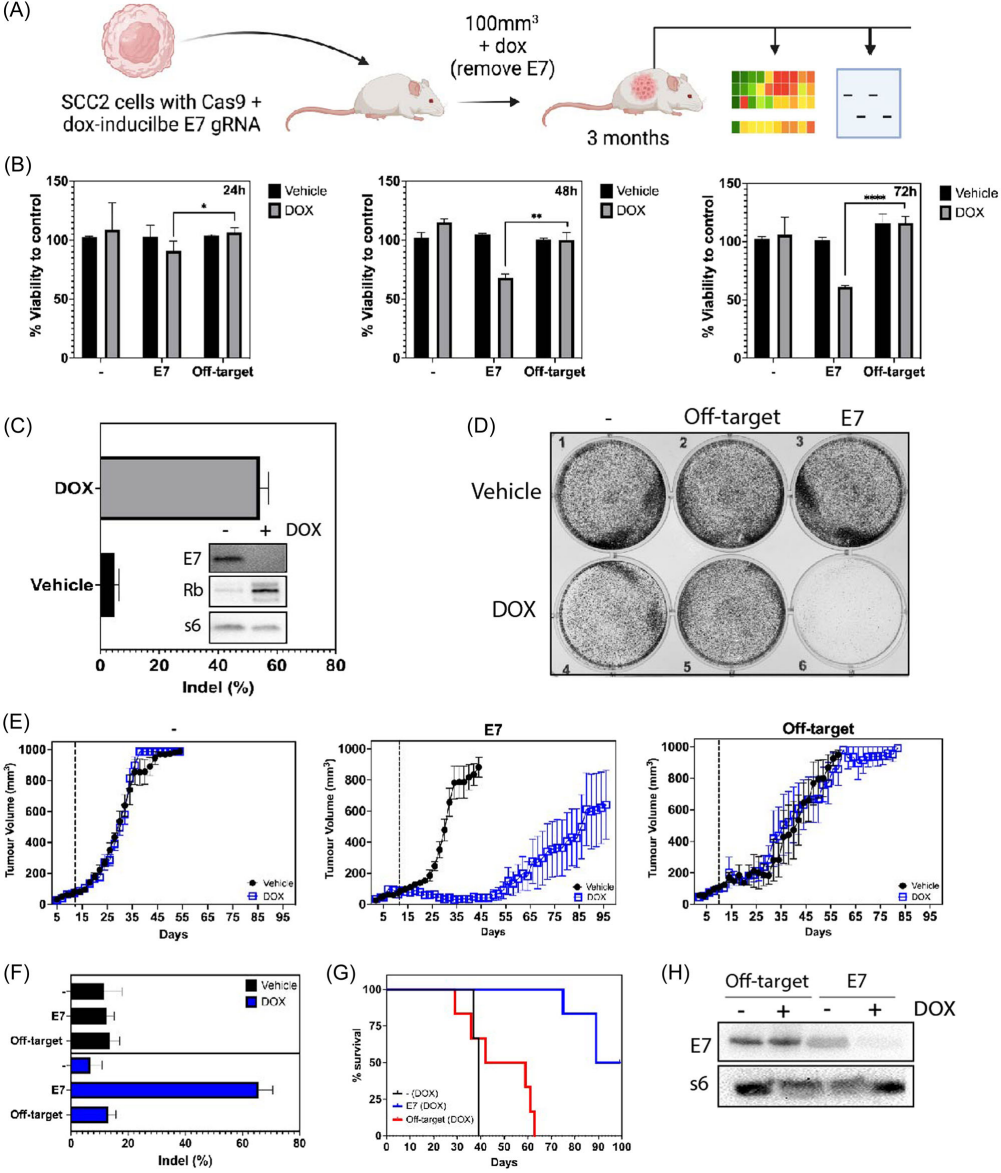
Genetic deletion of E7 results in effective amelioration of OPC tumor growth before recurring over time. (A) Schematic representation of the experimental plan. (B) SCC2_Cas9 (−), SCC2_Cas9_16E7 (E7) and SCC2_Cas9_18E7 (Off‐target) cells were either treated with sterile water (Vehicle) or with doxycycline (10 μg/ml) (DOX) before performing an MTT assay at indicated timepoints. Data representative of one out of three independent experiments. Bars denote mean percentage viability to SCC2_Cas9 control cells within its respective treatment groups. Error bars denotes standard error of mean (SEM) of technical quadruplicate treatments. The Student *t* test, *p = 0.0442; **p = 0.0015; ****p < 0.0001. (C) SCC2_Cas9_16E7 cells were either treated with sterile water (Vehicle) or with doxycycline (10 μg/ml) (DOX) for 72 h before extracting genomic DNA for Sanger sequencing. Percentage indel was determined by TIDE analysis (*n* = 3 independent cell treatments ± SEM). Proteins were also extracted from cells and immunoblotted for HPV 16 E7 and Rb. S6 protein was used as a loading control. Immunoblotting data is representative of one out of three independent experiments. (D) SCC2_Cas9 (−), SCC2_Cas9_16E7 (E7) and SCC2_Cas9_18E7 (Off‐target) cells were either treated with sterile water (Vehicle) or with doxycycline (10 μg/ml) (DOX) over 7 days before performing a colony forming assay. Data representative of one out of three independent experiments. (E) Nude mice were xenografted with either SCC2_Cas9 (−), SCC2_Cas9_16E7 (E7) or SCC2_Cas9_18E7 (Off‐target) cells and tumors allowed to grow to 100 mm^3^ before feeding mice with either normal (Vehicle) or DOX fodder (DOX). Each point represents mean of *n* = 5‐6 mice/group. Mean tumor volumes are shown with error bars representing SEM. (F) Tumors excised at indicated days (SCC2_Cas9 (−)—Day 37, SCC2_Cas9_16E7 (E7)—Day 89 and SCC2_Cas9_18E7 (Off‐target)—Day 37) were processed for genomic DNA extraction before performing Sanger sequencing. Percentage indel was determined by TIDE analysis (*n* = 6 tumors ± SEM). (G) Mouse survivorship (percentage survival) were evaluated and plotted throughout the experiment for DOX‐treated groups. (H) Proteins were extracted from tumors at indicated days (SCC2_Cas9 (−)—Day 37, SCC2_Cas9_16E7 (E7)—Day 89 and SCC2_Cas9_18E7 (Off‐target)—Day 37) and immunoblotted for HPV 16 E7. S6 protein was used as a loading control. Data representative of one out of three independent experiments, using tumors from different mice within its respective treatment groups. gRNA, guide RNA; OPC, oropharyngeal cancer

The human papilloma virus (HPV) is responsible for 5% of all human cancers, including 1/3 of global cases of oropharyngeal carcinomas (OPC).^7^ However, this rate can vary by geographical region from 0% to 85%. HPV^−^ OPCs are linked to alcohol and cigarette consumption, but HPV^+^ cancers are rising rapidly in young individuals.^8^ Genetic analysis reveals that HPV^+^ OPC cell lines and patient samples are almost universally p53 wildtype, due to the HPV E6 oncogene, while HPV^−^ OPC always contain mutated p53.^9^, ^10^ However, there are a small number of cases where p53 is mutant despite the presence of HPV. The question of whether this is an intermediate stage between HPV^+^ and HPV^−^ tumors has been raised by researchers, including ourselves. This provides a rationale for investigating if HPV^−^ tumors arise via a hit‐and‐run mechanism. This is further supported by data in HPV type 38 skin infections of mice, where deletion of viral oncogenes and tumor promotion with ultraviolet (UV) light results in tumors that no longer depend on the virus.^11^ Moreover, lower HPV gene expression results in phenotypical changes consistent with HPV^−^ OPC.^12^ Finally, in the HPV^+^ head and neck cell carcinoma cell line, 93‐VU‐147T, p53 is mutated,^9^ thus suggesting a lower reliance on viral oncogenes, and indeed the level of E6 and E7 is significantly lower in these cells.^13^ As such intermediate phenotypes have not been described for cervical cancers. This suggested to us that OPC may be less HPV oncogene addicted than cervical cancers and therefore afford us an opportunity to investigate if hit‐and‐run can occur in these cells. Moreover, the advent of new gene editing technologies allows us to mechanistically examine this at a biological level. To test this theory, we developed a novel HPV‐driven carcinogenesis model in which HPV type 16^+^ OPC SCC2 cells constitutively expressing the Cas9 protein (SCC2_Cas9)^14^ containing a doxycycline (DOX)‐inducible guide RNA (gRNA) would induce universal CRISPR‐driven disruption of the HPV E7 gene (Figure 1A). We selected the E7 oncogene as it is a major oncogenic driver in HPV cancers^15^ and because we had previously shown in HPV^+^ cervical cancers that removal of E7 eliminated tumors.^16^ We hypothesized that in HPV^+^ OPC, E7 removal would result in tumor clearance but that these would recur over time, having gained new genetic mutations to compensate for the loss of E7.

## 2 MATERIALS AND METHODS

### 2.1 Cells

The HPV type 16^+^ OPC cell line, UDSCC2 (SCC2), constitutively expressing Cas9 (SCC2_Cas9) were developed as previously described^14^ and primary mouse tonsil epithelial cells (MTECs)^17^ were obtained from Dr. Paola Vermeer (Sanford Research). Cells were maintained in complete media containing DMEM (Gibco‐Invitrogen) supplemented with 10% heat inactivated foetal bovine serum and 1% antibiotic/glutamine preparation (100 U/ml penicillin G, 100 U/ml streptomycin sulfate, and 2.9 mg/ml l‐glutamine) (Gibco‐Invitrogen). SCC2_Cas9 cells were maintained in complete media supplemented with blasticidin (InvivoGen) at 2 μg/ml. SCC2_Cas9 cells were generated by A/Prof Kaylene Simpson (Victorian Center for Functional Genomics, Peter Mac Cancer Center, Australia). Cas9 stable expressing cells lines were generated by transducing Cas9‐blasticidin vector (lentiCas9‐Blast; #52962; Addgene) at low multiplicity of infection (MOI) (~0.5). Cas9+ cells were then selected in blasticidin over 10–14 days and used as a polyclonal pool in all experiments. MTECs constitutively expressing Cas9 (MTEC_Cas9) were generated and maintained in a similar manner as SCC2_Cas9 cells.

### 2.2 Generating doxycycline inducible CRISPR system in SCC2_Cas9 and MTEC_Cas9 cells

CRISPick design tool was used to design the gRNAs targeting the HPV 16 E7 and 18 E7 oncogene and cross‐checked against other gRNA selection tools (ChopChop and Crispor). The HPV 16 E7 targeting gRNA (5′‐GCAAGTGTGACTCTACGCTT‐3′), HPV 16 E6 targeting gRNA (5′‐CCACTGTGTCCTGAAGAAAAGCA‐3′) and HPV18 E7 targeting gRNA (5′‐CCGGTTGACCTTCTATGTCA‐3′) were then cloned into the doxycycline (DOX)‐inducible gRNA expression plasmid, FgH1tUTG (#70183; Addgene),^18^ and confirmed by Sanger sequencing (BigDye Terminator v3.1 Cycle Sequencing Kit; Applied Biosystems 2002) before lentivirally infecting SCC2_Cas9 and MTEC_Cas9 cells. Cells were then purified and sorted on the BD FACSAria™ III Cell Sorter (BD Bioscience).

### 2.3 Chemicals and animal fodder

To promote single guide RNA expression in cell lines, doxycycline hyclate (DOX) (#D9891; Sigma‐Aldrich) was added to cell culture media at a final concentration of 10 µg/ml. Standard rodent food was supplemented with DOX (600 mg/kg body weight) (DOX fodder) for in vivo work (#SF08‐026; Specialty Feeds).

### 2.4 Cell viability determination

Cell viability was determined using the 3‐(4,5‐dimethylthiazol‐2‐yl)−2,5‐diphenyltetrazolium bromide (MTT) assay. MTT reagent (Sigma Aldrich) was added into cell media at a final concentration of 0.5 mg/ml for 2 h at 37°C before MTT crystals were dissolved in dimethyl sulfoxide to allow measurement of colorimetric absorbance at 544 nm using a FLUOstar OPTIMA microplate reader (BMG LabTech).

### 2.5 Colony forming assay

Cells were seeded at 50 000 cells per well in a 6‐well plate and left overnight to adhere before adding media containing 10 µg/ml of DOX. DOX‐supplemented media was changed daily over 7 days before media was removed and cells stained with crystal violet. Plates were visualized and imaged captured on a Chemidoc XRS Visualiser using a white light filter setting (BioRad).

### 2.6 SCC2_Cas9 and MTEC_Cas9 in vivo models

Female nude (aged 6–8 weeks) and C57BL/6 mice (aged 4 months) were purchased from the animal resource center, Perth, Australia. All animal experiments were performed in accordance with the Australian and New Zealand Council for the Care and Use of Animals in Research standards and were approved by the Griffith University Animal Ethics committee (MHIQ/14/20). Tumors were established by subcutaneously injecting 2 × 10^6^ respective SCC2_Cas9 cells (in nude mice) or MTEC_Cas9 cells (in C57BL/6 mice) (100 μl/injection in 50% PBS:50% Corning® Matrigel® solution (vehicle) (Sigma‐Aldrich) in the right flank of mice as done previously.^13^ Mice were monitored for tumor growth and when the tumors reached a size of approximately 100 mm^3^, mice were fed DOX fodder to induce the DOX‐inducible CRISPR system as previously described.^18^ Tumors volumes were measured using digital callipers every 2 days and mice health monitored daily. Mice were euthanized and culled at the end of designated monitoring period or when tumor mass reached 1000 mm^3^ or reached clinical endpoint (>15% weight loss, lack of grooming, reduce activity and appetite). Tumors were harvested and processed for immunohistochemistry staining using a p16 mouse monoclonal antibody (MAB 4133).

### 2.7 Time‐lapse microscopy

Cells were followed by time‐lapse microscopy using Holometer®, a cell stain‐free phase holographic imager (PHI AB; Lund) at 37°C and 5% CO_2_ and data analysed in Hstudio 2.7.5™ (PHI AB; Lund) on 24‐well plates (STARSTED).

### 2.8 Immunoblotting

Tumor proteins were extracted using AllPrep DNA/RNA/Protein Kit (#80004; QIAGEN), according to manufacturer's protocol. Immunoblots were probed with antibodies against HPV 16 E7 (NM2) (Santa Cruz Technologies Biotechnologies), Rb (Santa Cruz Technologies Biotechnologies) and S6 (Cell Signaling Technologies). Rabbit and mouse secondary antibodies (Cell Signaling Technologies) and ECL was used to detect protein signals on a Chemidoc XRS Visualiser (BioRad).

### 2.9 Determining gene editing efficiency

Cell genomic DNA was isolated using the QIAamp DNA Mini Kit (#51306; QIAGEN) according to manufacturers' instructions. Tumor DNA and RNA were extracted using AllPrep DNA/RNA/Protein Kit (#80004; QIAGEN), according to manufacturers' protocol. The HPV 16 E7 gene was amplified by PCR using primers previously designed^13^ and purified by ultrafiltration before performing Sanger sequencing using the BigDye Terminator v3.1 Cycle Sequencing Kit, Applied Biosystems 2002 (Part# 4337035A) following manufacturers' protocol. Sanger sequencing results were analysed using the online tool, TIDE (Tracking Indels by DEcomposition https://tide.nki.nl) as previously described.^19^

### 2.10 Whole exome sequencing (WES) and bioinformatics analysis

DNA samples were prepared for sequencing using the Twist Enzymatic Fragmentation (Twist Biosciences) preparation followed by Twist Exome V2 exome capture (Twist Biosciences) at the Australian Genome Research Facility (AGRF). Subsequently, libraries were sequenced on the NovaSeq. 6000 (Illumina) at the AGRF using a paired end 150 bp sequencing configuration. The FASTQ data were then assessed for quality and contamination before undergoing the somatic variant calling workflow at the AGRF. Briefly, each sample was aligned to the human consensus sequence (hg38) using the DRAGEN Bio‐IT platform v3.9.3 (Illumina). Reads identified as PCR or optical duplicates were then marked. Somatic small variant and indel calling was performed using DRAGEN v3.9.3 (Illumina) in a tumor/normal mode with each tumor sample compared against the SCC2 parental cell line. Variant calling was performed within the targeted capture region and included a “G/T” orientation bias filter corresponding to 8‐oxoguanine formation caused by oxidation. The resulting variant calls were annotated with the Variant Effect Predictor (VEP) v105.0.^20^

### 2.11 Pathway enrichment analysis

Pathway enrichment analysis was performed to identify pathways enriched in the selected gene list. Enrichr^21^ was used to perform pathway enrichment analysis, which produces Benjamini‐adjusted *p* value, *z* score, and total enrichment score (which incorporates both *p* value and *z* score information) for each pathway. Kyoto Encyclopedia of Genes and Genomes (KEGG) was selected as reference pathway database.

### 2.12 Statistical analysis

All statistical analyses were performed using the statistical software package GraphPad Prism v9 and described in detail in respective figure legends.

## 3 RESULTS AND DISCUSSION

Consistent with our previous observations,^22^ DOX treatment of SCC2_Cas9 cells bearing the HPV type 16 E7 targeting gRNA (SCC2_Cas9_E7) resulted in cell killing in a time‐dependent manner, with maximal killing capacity observed at 72 h when compared to the off‐target control (SCC2_Cas9 cells bearing the HPV type 18 E7 targeting gRNA) (Figure 1B, Videos S1, S2, and S4). The observed effect is consistent with the high E7 gene editing efficiency, loss of E7 and regained retinoblastoma (RB), a direct target of E7,^15^ protein expression at that time point (Figure 1C). In parallel, a colony forming assay revealed an almost complete loss of cell proliferation 7 days after cells were treated with DOX (Figure 1D). Importantly, genetic deletion of the E6 oncogene, another major oncogene essential for cervical cancer survival,^16^ had no effect on cell growth (Figure S1 and Video S3) suggesting that E7 is the critical oncogenic driver in SCC2 cell growth.

Having established the gene editing system, we next investigated the impact of genetic targeting of E7 in vivo over time. Using the same DOX‐inducible CRISPR system employed in this study, we previously showed genetic deletion of HPV E7 in SCC2 tumors resulted in effective tumor regression in vivo 20 days after DOX treatment.^22^ In this study, we plan to explore the possibility that these regressed E7‐edited tumors might recur over time. Nude mice bearing respective SCC2 tumors were treated with DOX in their food once tumors had reached 100 mm^3^ in volume. Compared to the off‐target group tumors, DOX‐treated SCC_Cas9_E7 tumors underwent efficient remission before progressively recurring from day 50 onwards (Figure 1E), whilst maintaining a survival advantage over the control (SCC_Cas9) and off‐target groups (Figure 1G). Importantly, the recurred tumors maintained a high E7 gene editing efficiency (Figure 1F), had lost E7 protein expression (Figure 1H) and reduced immunohistochemical p16 staining (Figure S2), a surrogate marker for HPV infection.

**Figure 2:**
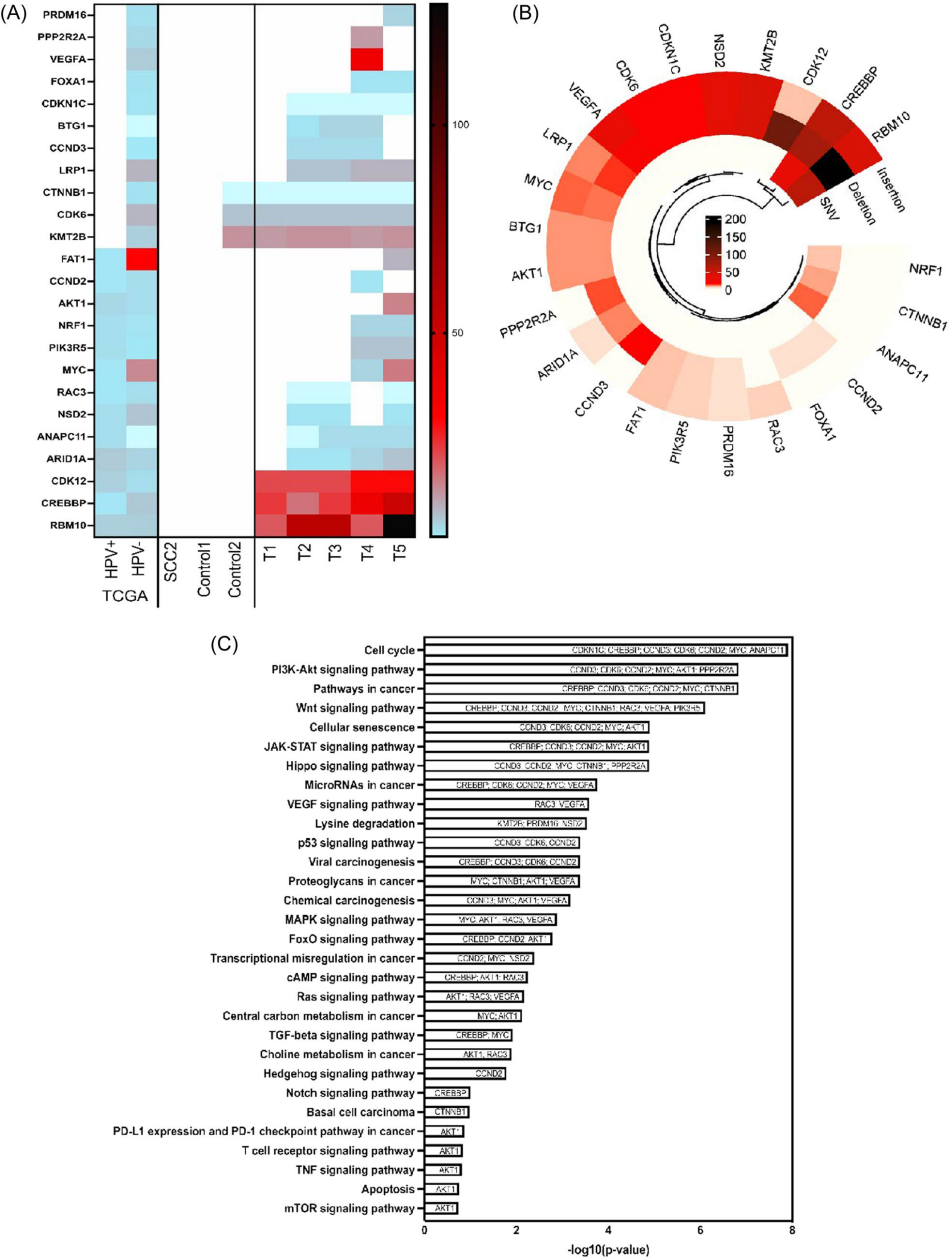
Recurred OPC tumors have gained novel mutations not previously present in HPV^+^ OPC tumors. (A) Heatmap of significantly mutated genes, corresponding to genes mutated only in HPV‐ samples or E7‐edited mice (DOX‐treated SCC2_Cas9 [control] and SCC2_Cas9_E7 [treatment]) and/or genes associated with cellular proliferation. For comparison, the mutation percentage of 302 HPV^−^ and 58 HPV^+^ head and neck squamous cell carcinoma (HNSCC) patients were compiled from the TCGA. The scale color bar represents the number of mutations encountered in each gene or the percentage of patients exhibiting mutation. Genes mutated in the SCC2 parental cell line were bioinformatically filtered and excluded. (B) Circular heatmap of type of mutations in the gene set. Combined number of SNV (gain or loss of stop codon), frameshift insertion or deletion for each examined gene. (C) The top 30 relevant enriched pathways for carcinogenesis. Plot showing −log‐transformed *p* value of enriched KEGG pathways for the 24 selected genes. KEGG, Kyoto Encyclopedia of Genes and Genomes; OPC, oropharyngeal cancer; SNV, single nucleotide variation; TCGA, The Cancer Genome Atlas

We next sought to understand what was driving the reoccurrence of these tumors in the absence of E7. It is well known that RB proteins^15^ regulate G1 phase progression in cells completing mitosis and in quiescent cells. The G1 cyclin‐dependent kinases phosphorylate phospho‐RB (pRB) proteins, thus facilitating the transition to S phase.^23^ In view of this, to overcome the presence of active pRB and maintain tumor progression, we predicted that relapsed tumors lacking E7 would develop mutations that critically impact cell cycle regulation pathways. Remarkably, WES revealed many novel mutations in relapsed tumors that are not present in the parental cell line (Table 1). Indeed, 24 genes associated with cell cycle regulation and/or transcriptional regulation were identified as being substantially altered in recurred E7‐edited tumors (Figure 2A), either as single nucleotide variation (gain or loss of stop codon), frameshift insertion, or deletion mutations (Figure 2B). Importantly, 11 of these mutated genes matched common mutations in HPV^−^ patient samples from The Cancer Genome Atlas data but were not observed in HPV^+^ OPC patient tumors, nor in control tumors (Figure 2A). These data indicate a shared solution to the loss of E7 between HPV− patient tumors and recurring tumors in our system and strongly support a hit‐and‐run mechanism as the origin of these tumors. Pathway analysis of these mutations further confirm this, showing the mutations disrupt important pathways intricately associated with cell proliferation (Figure 2C). The top enriched KEGG pathway for the selected genes includes cell cycle PI3K‐Akt signaling pathway, Wnt signaling pathway, JAK‐STAT signaling pathway, HIPPO signaling pathway and p53 signaling pathway (*p* < 0.01) and have roles in key pathways involved in carcinogenesis.

**Table 1:** DOX‐treated SCC2_Cas9 (control) and SCC2_Cas9_E7 (treatment) tumors subjected to whole exome sequencing (WES), showing tumor volumes, culling days and total number of genes significantly mutated

Group/mouse	Tumor volume (mm^3^)	Culling day	# Genes mutated
Control 1	1000	37	12
Control 2	1000	57	34
Treatment T1	52.8	31	71
Treatment T2	1000	75	98
Treatment T3	461	100	195
Treatment T4	166	89	151
Treatment T5	1000	89	53

Overall, these findings show that HPV facilitates the accumulation of mutations in OPC but in the absence of E7 tumors quickly acquire novel mutations that ensure ongoing cell division. Thus, viral oncogenes initiate the development of cancer, but we showed they are clearly no longer necessary after the tumor has gained necessary mutations. Therefore, a transient infection by HPV (“hit”) may induce gene expression modifications that would be propagated over many cell divisions, even after the clearance of the virus (“run”). There are several likely scenarios to how this may occur. In a similar manner to that proposed for several oncogenic viruses,^24^ HPV could evade cellular defences by engaging directly with epigenetic machinery, therefore deregulating various important host genes and pathways. HPV may also deregulate the gene expression pathways operating in hit‐and‐run oncogenesis in oral mucosa via interaction between viral proteins and host proteins involved in regulation of transcriptional program.^25^ HPV‐encoded proteins may also interact with cellular proteins that protect the genome from mutations, which may stably silence or upregulate key cellular genes involved in the regulation of cell proliferation and genomic stability. The identification and characterization of putative pathways dysregulated in HPV‐mediated hit‐and‐run oncogenesis could aid understanding of the mechanism of oral carcinogenesis. In turn, this could serve as the foundation for the establishment of biomarkers that may be utilized for the monitoring of individuals at greater risk and the implementation of preventative interventions aimed at reversing early crucial molecular processes leading to the development of cancer. There are several limitations in this work. First, work presented here is representative of a single HPV^+^ OPC cell line. Given that other HPV^+^ OPC cells, SCC104, SCC90, and 147T, were affected to the same degree as SCC2 cells with RNAi‐mediated E7 knockdown in vitro,^13^ we predict that loss of E7 by CRISPR would result in a similar observation in vivo (i.e., initial tumor regression and later recurrence). The HPV^+^ OPC in vivo model used here is in an immunocompromised host (nude mice). Hence, any contribution of the adaptive immune system on tumor recurrence cannot be examined. We explored the possibility of performing these experiments in an immunocompetent mouse (C57BL/6) using primary MTECs engineered to express HPV E7, E6 and Ras (MTECs).^17^ Indeed, MTEC_Cas9_E7 cells treated with DOX had reduced cell viability (Figure S4), consistent with what we observe in SCC2 cells (Figure 1B). However, these tumors only grew transiently and were unable to sustain growth over a long period of time (Figure S3), rendering this model experimentally unsuitable for testing our hypothesis.

To our knowledge, this is the first animal model using a reliable CRISPR/Cas9 platform to efficiently knockdown the HPV E7 oncogene for OPCs, enabling the study of mutations in relapsed tumor in the absence of a functional E7 protein. Our data implies that we may be underestimating the load of HPV‐driven OPC as many HPV^−^ malignancies may have originated as HPV^+^ tumors that have lost HPV. At this time, it is unclear how such a loss may occur. It also has important implications for HPV vaccination efforts as it would suggest that HPV vaccination would not only reduce HPV^+^ OPC, but also HPV^−^ OPCs. Universal HPV vaccination of males, who are over‐represented in OPC cancer, only began in 2011 in some countries, such as Australia, and therefore reduced OPC would be expected to occur when these patients reach 40 years of age and above.

## AUTHOR CONTRIBUTIONS

**Danyelle Assis Ferreira**: conducted the experiments, generated experimental data, performed data analysis, and drafted the manuscript. **Adi Idris and Nigel A. J. McMillan**: conceived and designed the work, performed detailed data analysis, interpreted the data, ensured the integrity of the data presented and drafted the manuscript to its final form. All authors approved the final version of the manuscript.

## CONFLICTS OF INTEREST

NAJM receives payment from UniQuest Pty Ltd. and is also a consultant for Prorenata Ltd.

## ETHICS STATEMENT

All animal experiments were performed in accordance with the Australian and New Zealand Council for the Care and Use of Animals in Research standards and were approved by the Griffith University Animal Ethics committee (MHIQ/14/20).

## Supporting information

Supplementary information.

Supplementary information.

Supplementary information.

Supplementary information.

Supplementary information.

Supplementary information.

Supplementary information.

Supplementary information.

Supplementary information.

## Data Availability

The data that supports the findings of this study are available in the Supporting Information Material of this article All data generated or analysed during this study are included in this manuscript.
