# Supplementary figures and images for: Analysis of a hit‐and‐run tumor model by HPV in oropharyngeal cancers
Source: J Med Virol. 2022 Nov 4;95(1):e28260. doi: 10.1002/jmv.28260 (PMC9828080; doi:10.1002/jmv.28260)

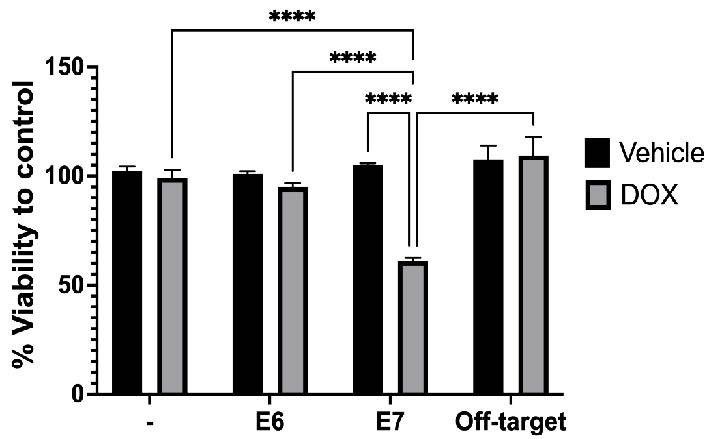

Supplement: Supplementary file 1 — Supplementary information. [file JMV-95-0-s002.jpg]

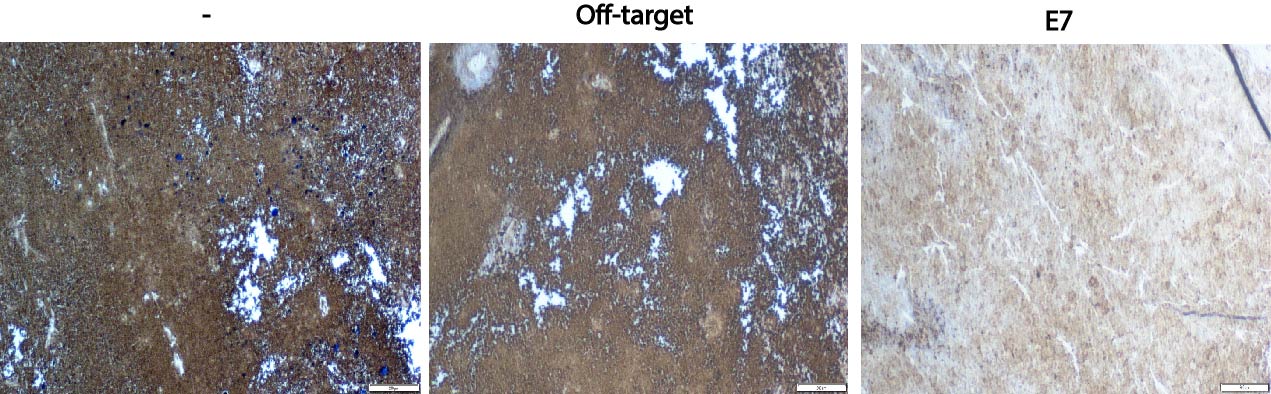

Supplement: Supplementary file 2 — Supplementary information. [file JMV-95-0-s004.jpg]

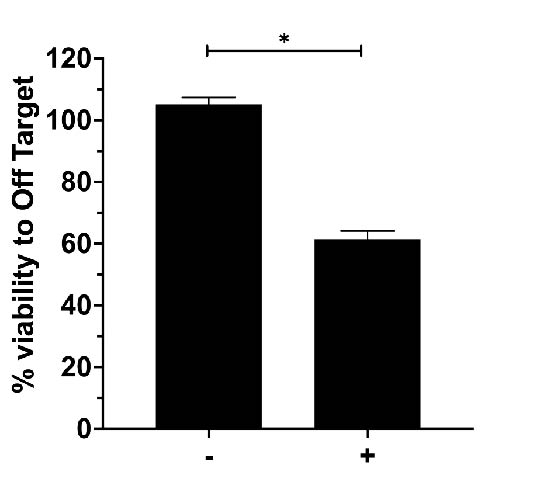

Supplement: Supplementary file 3 — Supplementary information. [file JMV-95-0-s008.jpg]

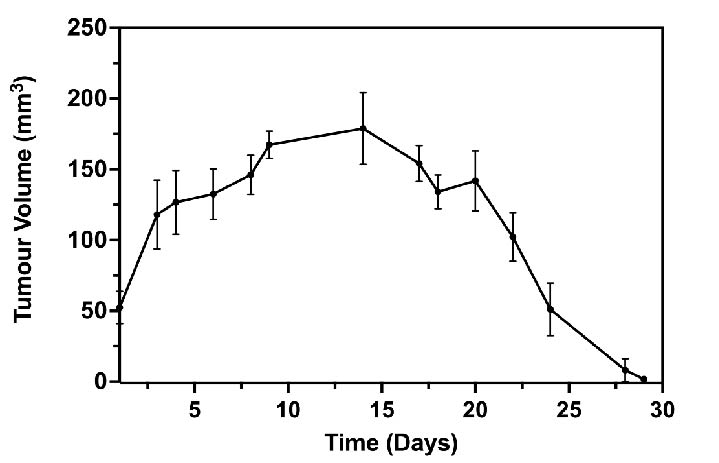

Supplement: Supplementary file 4 — Supplementary information. [file JMV-95-0-s005.jpg]
