# Supplementary material for: Analysis of a hit‐and‐run tumor model by HPV in oropharyngeal cancers
Source: J Med Virol. 2022 Nov 4;95(1):e28260. doi: 10.1002/jmv.28260 (PMC9828080; doi:10.1002/jmv.28260)
Supplement: Supplementary file 9 — Supplementary information. [file JMV-95-0-s007.docx]

**Supplementary videos**

**1)** SCC2_Cas9 (-), **2)** SCC2_Cas9_16E7 (E7), 3) SCC2_Cas9_16E6 (E6) and 4**)** SCC2_Cas9_18E7 (Off-target) cells were treated with doxycycline (10μg/ml) (DOX) for 96h and subjected to live cell imaging on the Holometer®.

**Supplementary figures**

**Supplementary figure 1** SCC2_Cas9 (-), SCC2_Cas9_16E7 (E7), SCC2_Cas9_16E6 (E6) and SCC2_Cas9_18E7 (Off-target) cells were either treated with sterile water (Vehicle) or with doxycycline (10μg/ml) (DOX) before performing an MTT assay at 72h. Data representative of one out of three independent experiments. Bars denote mean percentage viability to SCC2_Cas9 control cells within its respective treatment groups. Error bars denotes SEM of technical quadruplicate treatments. Student’s T-test, **** p<0.0001.

**Supplementary figure 2** Immunohistochemical staining of representative tumour tissues from DOX-treated groups at indicated days (SCC2 Cas9_16E7 (E7) – Day 89 and SCC2 Cas9_18E7 (Off-target) – Day 37) with a p16 antibody and imaged at 4X magnification. Data representative of tumour tissues from one out of five mice. Scale bar = 200μm.

**Supplementary figure 3** MTEC_Cas9_16E7 (E7) and MTEC_Cas9_18E7 (Off-target) cells were either treated with sterile water (-) or with 10μg/ml doxycycline (+) before performing an MTT assay at 72h. Bars denote mean percentage viability to MTEC_Cas9_18E7 cells (Off-target) within its respective treatment groups. Error bars denotes SEM of technical quadruplicate treatments. Student’s T-test, * p=0.0286.

**Supplementary figure 4** 2x10^6^ MTEC_Cas9 cells were implanted into C57BL/6 mice and tumours allowed to grow. Tumour measurements began seven days after implantation (Day 0) over a 30-day period. Each point represents mean of n = 6 mice/group. Mean tumour volumes are shown with error bars representing SEM.
